# Methyltransferases acquired by lactococcal 936-type phage provide protection against restriction endonuclease activity

**DOI:** 10.1186/1471-2164-15-831

**Published:** 2014-10-01

**Authors:** James Murphy, Jochen Klumpp, Jennifer Mahony, Mary O’Connell-Motherway, Arjen Nauta, Douwe van Sinderen

**Affiliations:** School of Microbiology, University College Cork, Cork, Ireland; Institute of Food, Nutrition and Health ETH Zurich, Zurich, Switzerland; Alimentary Pharmabiotic Centre, University College Cork, Cork, Ireland; FrieslandCampina, Amersfoort, The Netherlands; School of Microbiology, National University of Ireland, Room 4.05 Western Road, Cork, Ireland

**Keywords:** *Lactococcus lactis*, Bacteriophage, Methylome, Restriction-modification, SMRT sequencing

## Abstract

**Background:**

So-called 936-type phages are among the most frequently isolated phages in dairy facilities utilising *Lactococcus lactis* starter cultures. Despite extensive efforts to control phage proliferation and decades of research, these phages continue to negatively impact cheese production in terms of the final product quality and consequently, monetary return.

**Results:**

Whole genome sequencing and *in silico* analysis of three 936-type phage genomes identified several putative (orphan) methyltransferase (MTase)-encoding genes located within the packaging and replication regions of the genome. Utilising SMRT sequencing, methylome analysis was performed on all three phages, allowing the identification of adenine modifications consistent with N-6 methyladenine sequence methylation, which in some cases could be attributed to these phage-encoded MTases. Heterologous gene expression revealed that M.Phi145I/M.Phi93I and M.Phi93DAM, encoded by genes located within the packaging module, provide protection against the restriction enzymes HphI and DpnII, respectively, representing the first functional MTases identified in members of 936-type phages.

**Conclusions:**

SMRT sequencing technology enabled the identification of the target motifs of MTases encoded by the genomes of three lytic 936-type phages and these MTases represent the first functional MTases identified in this species of phage. The presence of these MTase-encoding genes on 936-type phage genomes is assumed to represent an adaptive response to circumvent host encoded restriction-modification systems thereby increasing the fitness of the phages in a dynamic dairy environment.

**Electronic supplementary material:**

The online version of this article (doi:10.1186/1471-2164-15-831) contains supplementary material, which is available to authorized users.

## Background

The bacterio(phage) – host arms race represents a dynamic interplay of survival among a population of bacteria and their infecting viral parasites [1]. Depending on the complexity of the environment, the ongoing antagonistic evolution can generate diverse populations among both phages and their bacterial hosts [2–4]. Host adaptation is driven by the highly selective pressure of lytic phages, while phages are in turn compelled to mutate in order to achieve efficient host infection, combined with, in the case of virulent phages, optimal production and release of progeny particles [5]. An example of such adaptive interplay are phages that modify their receptor binding protein (RBP) or tail fibres to target a new cell surface receptor if the original receptor becomes unavailable as seen in *Escherichia coli* phage cI26 [6, 7]. Bacterial genomes and plasmids may encode a wide variety of defence mechanisms to combat phage infection, such as restriction-modification systems (R-Ms), abortive infective (Abi) systems and CRISPR-mediated immunity [8, 9]. Nonetheless, phages have been shown to be able to bypass many of these phage-resistance systems in order to successfully continue their replication and proliferation. For example, phages may evade CRISPR systems by acquiring mutations in the protospacer, thus preventing complementary binding of the CRISPR-produced crRNA to the target phage DNA [10]. Furthermore, phage genomes have been shown to acquire methyltransferase (MTase)-encoding genes, which are termed orphan MTases if they occur in the absence of their cognate restriction enzyme-encoding gene [11]. Their function is to actively methylate phage DNA to negate the activity of host encoded restriction endonucleases which recognize the same sequence. MTase-encoding genes are found on the genomes of, among others, T-even phages of *E. coli*, several *Bacillus subtilis* phages, and the lactococcal phi-50 (P335-type phage) [12–14]. In some cases phages have been shown to specify complete R-M systems as observed in the *Staphylococcus aureus* quadruple converting (causes lysogenized bacteria to acquire or lose the ability to express phenotypic traits) phage π42, which harbours a BcgI-like R-M system [15].

In the dairy industry, selection of phage-resistant starter cultures coupled with extensive phage control strategies may reduce the risk of phage infection of hosts and decrease their ability to engage in antagonistic evolution [16–18]. However, some examples of 936-type phages overcoming host-encoded systems include mutations in the *sak* and *sav* genes, which allow such mutated phages (referred to as escape mutants) to circumvent the abortive infection systems AbiK [19, 20] and AbiV, respectively [21]. Most recently it has been demonstrated that certain mutations in the gene specifying the major capsid protein allow the 936-type phage sk1 to overcome the AbiB system of *L. lactis* UC509.9 [22]. Previously, we reported on the isolation of phages from a mixed starter system [23], and showed that, consistent with earlier surveys [24, 25] members of the 936-type phages are the only detected phages within the examined fermentation facilities. The reasons behind the persistence and prevalence of the 936-type phages are undoubtedly multifactorial and encoded by their genome, which encompasses many genes without an assigned function. The 936-type phage genomes, with sizes ranging from 26–32 kb, are modular in organisation and are clustered into late, early and middle-expressed genes, with the early transcript encompassing the largest number of genes with unknown function [26]. The advances in sequencing technology and the variety of sequencing platforms available has allowed for a significant increase in the number of fully sequenced phage genomes [27]. Continued phage isolation and rapid genomic characterization is crucial in order to unravel the underlying reasons and mechanisms for the occurrence and persistence of particular phage species, especially 936-type phages in lactococcal fermentations. Here, we report on the genome sequences of three 936-type phages, Phi93, Phi145 and Phi15, and for the first time show that the 936-type phages can acquire (orphan) MTases which provide a protective effect against specific restriction endonuclease activities.

## Results and discussion

### Identification of 936-type phages encoding putative (orphan) methyltransferases

**Figure 1:**
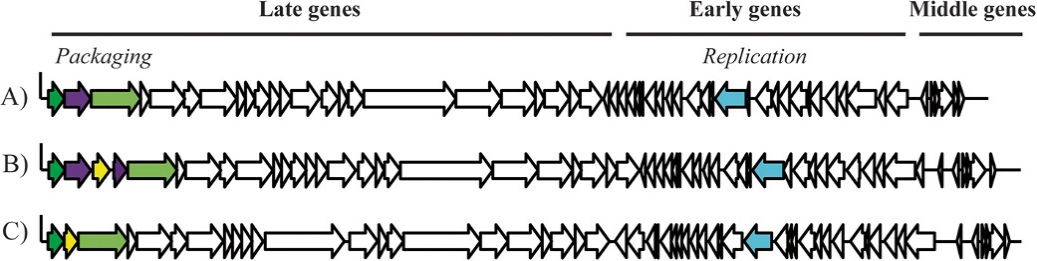
**Schematic representation of the 936-type phage genome. A)** Phi145 **B)** Phi93 and **C)** Phi15. green arrow symbol= Small terminase, yellow-green arrow = Large terminase, violet arrow symbol = Putative MTase (*mtPhi145-1, mtPhi93-1* and *mtPhi93-DAM*). yellow arrow symbol = Homing endonuclease and sky blue arrow symbol = Putative MTase (*mtPhi145-2*, *mtPhi93-2* and *mtPhi15-1*).

**Figure 2:**
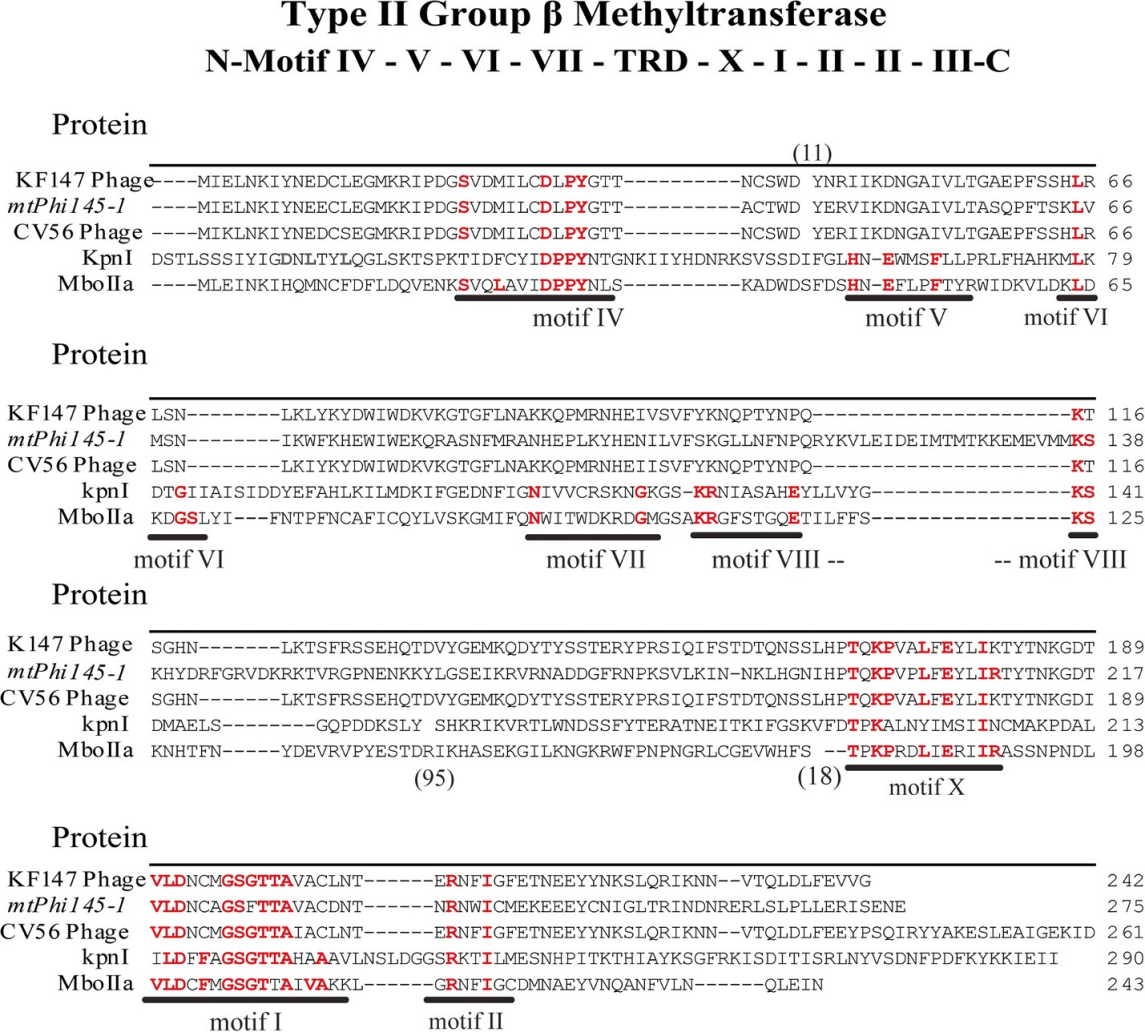
**Type II group β MTases.** Protein alignment of the putative (orphan) MTases of Phi145, *mtPhi145-1*, the MTases encoded by prophage CV56 (YP_005868377) & KF147 (YP_0033553511) and the representative MTases MboIIa (P23192) and KpnI (P25238). The group β MTases conserved motifs previously determined [30, 31] are underlined and conserved residues are highlighted in red bold letters. To aid in the identification of conserved motifs some residues were removed from the alignment and indicated by the numbers in parenthesis: (11) = 11 amino acids removed from KFI47, CV56 and *mtPhi145-1*; (95) = 95 amino acids removed from KpnI and (18) = 18 amino acids as described previously and (18) = 18 amino acids removed from MboIIa as described previously [30, 31].

Initial genome sequencing was performed on a 454 device on phage DNA isolated from three lactococcal 936-type phages (Phi93, Phi145 and Phi15), previously isolated from whey samples obtained from Gouda-producing cheese factories (Table 1) [23]. The genomic characteristics of the three phages are summarized in Table 2. The three genomes each encompass 55 ORFs (Additional file 1: Table S1), apparently organised into three transcriptional modules (as based on gene orientation), a gene arrangement that is typical for 936-type phages (Figure 1) [26, 28]. Typically, the consensus gene order of the packaging module consists of the gene encoding the putative small terminase subunit followed by that specifying the large terminase subunit as seen, for example, in the genomes of jj50, sk1 and P008 [26]. However, this region appears to be a hotspot for genetic insertions and several 936-type phages were observed to possess an additional ORF of unknown function located downstream of the gene encoding the small terminase subunit [25, 28]. Annotation of the genomes revealed that also the genomes of Phi15, Phi93 and Phi145 each contain additional ORFs in this region of their genomes (Figure 1), including ORFs that specify putative (orphan) MTases (Summarised in Table 3). In the case of Phi15 the deduced protein product of locus tag Phi15_02 was predicted to specify a homing endonuclease (HNHE), while for Phi145, the similarly positioned gene, designated here as *mtPhi145-1* (Nomenclature assigned according to Roberts et al., 2003 [29]) (corresponding to locus tag Phi145_02) (Figure 1) is predicted to encode a putative (orphan) MTase, and accordingly named M.Phi145I (Nomenclature of the identified MTases was according to Roberts et al., 2003 [29]) (Table 3). The Phi93 genome has three additional ORFs located between the genes that encode the putative large and small terminase subunits: *mtPhi93-1*, *HNHPhi93-3* and *mtPhi93-DAM* (corresponding to locus tags Phi93_02, Phi93_03 and Phi93_04, respectively), which are predicted to specify an MTase, accordingly named M.Phi93I, a HNHE, designated as *PHE*asePhi93I (*p*utative *h*oming *e*ndonuclease), and a DAM MTase, named M.Phi93DAM, respectively (Figure 1) (Table 3). Using BlastP and HHpred analyses, M.Phi145I and M.Phi93I, whose amino acid (aa) sequences share 99% similarity, were found to share sequence similarity (50 aa% identity) to the prophage MTases of *L. lactis* CV56 (GenBank: YP_005868377) and KF147 (GenBank:YP_003353511), and 22% aa identity to the MTase MboIIa (GenBank: P23192). Amino acid alignments of the above-mentioned putative MTases with MboIIa and KpnI (GenBank: P25238) identified several of the motifs associated with methyltransferases, and based on the order they occur (III, IV, VI, VII, VIII, X, I, II), M.Phi145I and M.Phi93I are believed to belong to the type II-encoding genes, group β MTases (Figure 2) (Table 3) [30, 31].

**Table 1:** **Bacteria, phages, plasmids and primers used in this study**

Bacteria	Features	Source
*L. lactis* NZ9000	Host strain for expression	[53]
*L. lactis* SM M	Host strain for Phi93	[23]
*L. lactis* SM E	Host strain for Phi145	[23]
*L. lactis* SM 13	Host strain for Phi15	[23]
*L. lactis* SM 11	2^nd^ propagating strain for Phi15	[23]
*E. coli* EC101_pPTPi	Strain carrying low-copy plasmid pPTPi	[23]
*E. coli* EC101	Host strain for cloning	[54]
*E. coli* K12	Competent, *DAM* ^*-*^ */DCM* ^*-*^ Strain for pPiM.93DAM cloning	NEB
**Phage**	**Features**	**Source**
Phi15	Methylase positive^a^	[23]
Phi93	Methylase positive	[23]
Phi145	Methylase positive	[23]
**Plasmids**	**Features**	**Source**
pPTPi	Tetracycline resistant, Nisin inducible, ^b^Tet^r^	[52]
pPiM.93DAM	pPTPi derivative encoding *mtPhi93-DAM*, ^b^Tet^r^	This study
pPiM.145I	pPTPi derivative encoding *mtPhi145-1*, ^b^Tet^r^	This study
pPiM.145II	pPTPi derivative encoding *mtPhi145-2*, ^b^Tet^r^	This study
**Primers**	**Sequence (5'-3')** ^**c**^	**Source**
93DAMMTaseF	agcagc**GGATCC**AGGAGGCACTCACATGCACCATCATCATCATCATTCTTCTGGTAATAATGAATTAATG	This study
93DAMMTaseR	gctgct**CCCGGG**TTAATGATGATGATGATGGTGACCAGAAGTTCAAATATCACGACCATG	This study
Phi145.M1F	agcagc**GGATCC**aggaggcactcacATGCACCATCATCATCATCATTCTTCTGGTATTGAATTAAATAAA	This study
Phi145.M1R	agcagc**CCCGGG**TTACTATTCGTTTTCAGATAT	This study
Phi145.M2F	agcagc**GGATCC**aggaggcactcacATGCACCATCATCATCATCATTCTTCTGGTCTTAAGTTAGACGAG	This study
Phi145.M2R	agcagc**CCCGGG**TTAACTGTTTTTAACCATAAA	
mcsPTPiFwd	CTGAGGTTCTTATGGCTC	[51]
mcsPTPiRev	TTCGCTTTTAAAGTCGATTTCAT	[51]

^a^Predicted from BlastP and REBASE.

^b^Tet^r^?=?Tetracycline resistant.

^c^Restriction sites incorporated into oligonucleotide primer sequences are indicated in bold.

**Table 2:** **Summary of the characteristics of the sequenced 936-type phage**

Phage	Genome size (bp)	% G + C	No. of ORFs	***cos***sequence	Source
Phi15	31945	34.58	55	CACAAAGGACT	[23]
Phi93	31841	34.97	55	CACAAAGGTCT	[23]
Phi145	30862	34.90	55	CACAAAGGTCT	[23]

**Table 3:** **Summary of putative MTases in the 936-type phage**

Phage	Locus tag	Gene designation	Protein	Genome location	Target motif	Type	Group	Conserved motifs
Phi15	Phi15_36	*mtPhi15-1*	M.Phi15I	Replication	5′-CC^6m^AG-3′^a^	II	α	X, I, II, IV, VI, VIII
Phi93	Phi93_02	*mtPhi93-1*	M.Phi93I	Packaging	5′-GGWG^6m^A-3′^b^	II	β	IV, VI, X, I, II
	Phi93_04	*mtPhi93-DAM*	M.Phi93DAM	Packaging	5′-G^6m^ATC-3′^c^	II	-	IV
	Phi93_39	*mtPhi93-2*	M.Phi93II	Replication	5′-CY^6m^AG-3′^d^	II	α	X, I, II, IV, VI, VIII
Phi145	Phi145_02	*mtPhi145-1*	M.Phi145I	Packaging	5′-GGWG^6m^A-3′^b^	II	β	IV, VI, X, I, II
	Phi145_37	*mtPhi145-2*	M.Phi145II	Replication	5′-CY^6m^AG-3′^d^	II	α	X, I, II, IV, VI, VIII

^a^Motif identified on two independent SMRT sequencing runs with Phi15 propagated on two separate hosts.

^b^Validated by restriction analysis using HphI. Phi93_002 and Phi93_039 are 99% and 100% identical to Phi145_002 and Phi145_037 respectively, therefore only the genes from Phi145 were cloned and validated by restriction analysis.

^c^Validated by restriction analysis using DpnI and DpnII.

^d^Assigned based on comparative sequence alignment to *mtPhi15-1*.

HHpred analysis of M.Phi93DAM showed that this (orphan) MTase shares 63% aa identity to the *S. aureus* prophage L54a-encoded putative N-6 adenine MTase (GenBank: YP_185238.1). Using REBASE it was predicted that *mtPhi93-DAM* encodes a putative DAM MTase, recognising the motif 5′-GATC-3′, however, and in contrast to other DAM MTases, M.Phi93DAM was found to only harbour a single conserved MTase motif, Asn-Pro-Pro-Tyr (NPPY) [12].

**Figure 3:**
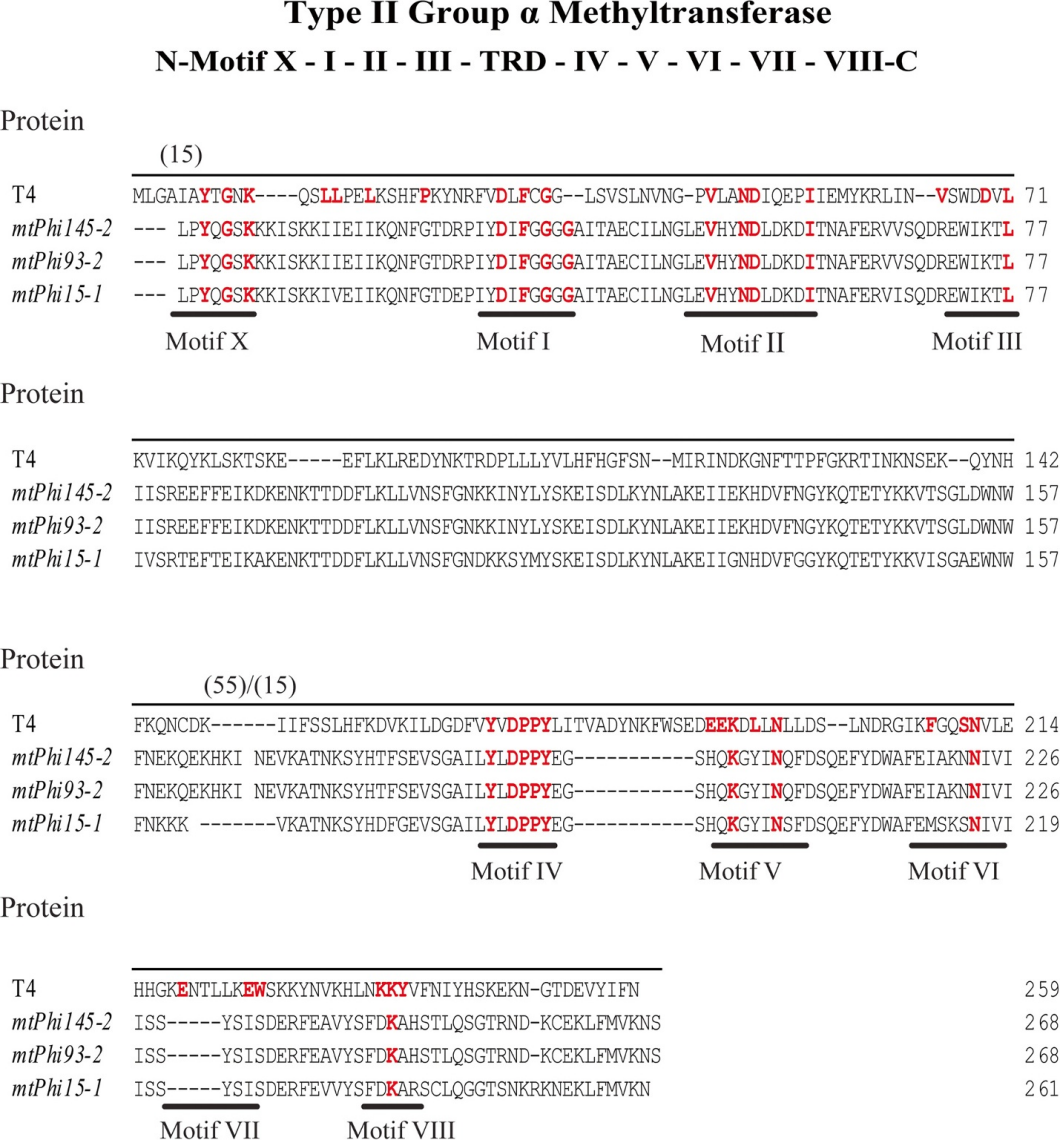
**Type II group α MTases.** Protein alignment of the putative phage (orphan) MTases located in the replication region: *mtPhi145-2*, *mtPhi93-2, mtPhi15-1* and T4 phage DAM MTase (NP_049647). The group α MTases conserved motifs previously determined [12, 30] are underlined and conserved residues are highlighted in red bold letters. To aid in the identification of conserved motifs some residues were removed from the alignment and indicated by the numbers in parenthesis: (15) = 15 amino acids removed from *mtPhi145-2*, *mtPhi93-2, and mtPhi15-1*; (55/15) = 55 amino acids removed *mtPhi145-2*, *mtPhi93-2* as well as 15 amino acids from *mtPhi15-1*.

Individual ORFs located within the replication regions of the Phi145, Phi93 and Phi15 genomes (corresponding to locus tags Phi145_37, Phi93_39 and Phi15_36) (Figure 1) (Table 3), designated here as *mtPhi145-2, mtPhi93-2,* and *mtPhi15-1*, respectively, were also found to encode proteins (M.Phi145II, M.Phi93II and M.Phi15I) predicted to specify (orphan) MTases based on BlastP, REBASE and HHpred analysis (Table 3). These putative (orphan) MTases are not unique to the phage sequenced in this study as they were also found to appear in several other 936-type phages such as ASCC191 (GenBank: AFE86771) and *Caseus*JM1 (GenBank: AGE60667) [28, 32]. Amino acid alignments with the *E. coli* T4 phage MTase (NP_049647) identified these ORFs as group α type II N-6 MTases based on the presence of several of the conserved motifs associated with this group and the particular order they occur in (X, I, II, III, IV, VI and VIII) (Figure 3). These three phage-encoded MTases lacked motif VII and had only one conserved residue for motifs III, VI and VIII [30, 33] (Figure 3). While all four MTases did not harbour all nine conserved MTase motifs, typically observed in group α type II N-6 MTases, variations in motifs have been seen before such as in HhaII (GenBank: P00473) in which motif IV is represented as DPQYR instead of N/DPPYN. Type II MTases are often associated with a cognate restriction endonucleases making up type II R-Ms in lactococcal strains which play an important role in protecting these strains from phage infection [22]. It has been demonstrated that lytic lactococcal phages have the ability to acquire functional MTases as shown for phi-50 which possesses the nucleotide sequence encoding an amino domain, LlaPI, from the R-M LlaI, identical to that on the plasmid pTR2030 [13]. It is believed that the MTases identified in this study are (orphan) MTases as they do not appear to be associated with a cognate restriction endonuclease which may have occurred due to the negative impact a restriction endonuclease may have on the phage DNA or that the amount of additional genomic information that can be acquired in the region of the phage genome may be limited, i.e. there is no selective advantage in acquiring the restriction endonuclease component.

Acquisition of additional ORFs by lytic phages may occur due to errors during phage DNA packaging, and appears to be more frequently encountered in *pac*-type phages, which use the head-full packaging mechanism, due to the recognition of pseudo*-pac* sites on the host DNA [34, 35]. However, packaging of additional DNA has also been shown for *cos*-type phages, such as 12 and SLT, which have been shown to mobilise *S. aureus* pathogenicity islands [36]. The observed sequence similarity between the packaging module-associated MTase-encoding genes with sequences located within the prophage elements of *L. lactis* KF147 and CV56 may indicate a genetic exchange event either between the phage and a prophage sequence within the host genome, or between phage genomes during co-infection with a replicating temperate phage via non-homologous recombination. Previous studies have shown that lactococcal strains encode type II R-M systems (LlaAI, LlaBI, LlaDCHI, and LlaKR21) specifying DpnI and DpnII isochizomers (5′-GATC-3′), and it is possible that Phi93 acquired *mtPhi93-dam* from a host harbouring such an R-M system [37, 38]. These packaging module-associated MTase-encoding genes appear to be unique to the 936-type phages sequenced in this study. MTases have been implicated in several functions in phages, primarily that of providing protection against host-encoded endonucleases [11, 39], yet regulatory roles have also been proposed for those associated with the packaging genes in *E. coli* phage P1 [40]. GATC methylation has been shown to be required to ensure efficient packaging of the phage DNA as loss of this methylation resulted in a reduction in progeny phage numbers. It is unlikely that the MTases identifed in this study fulfill a regulatory role, as there are no reported 936-type phages (prior to this study) that harbour (orphan) MTase-encoding genes between the large and small terminase-encoding genes. It is more plausible that the MTases represent an acquired defence whereby phage DNA is methylated such that it will be protected from endonuclease activity that may be present in prospective hosts.

### Epigenomic analysis of phage DNA reveals distinctive methylation profiles

To determine the methylation specificities of the predicted phage MTases, Phi93 (propagated on *L. lactis* strain SM M), Phi145 (propagated on *L. lactis* strain SM M and SM E in order to distinguish host-specific methylation patterns) and Phi15 (propagated on *L. lactis* strains SM 13 and on strain SM 11) were subjected to SMRT DNA sequencing [41, 42], a real-time approach that allows for the detection of modified nucleotides [6-methyladenine (6 mA), 5-methylcytosine (5mC) and 5-hydroxymethylcytosine (5hmC)] in the DNA sequence based on the DNA polymerase kinetics [42]. Several methods are available to study DNA methylation such as bisulphite treatment, HPLC, and microarrays, although it is challenging to detect 6 mA methylation patterns using any of these methods [43]. A certain minimum sequencing coverage is necessary for methylome analysis and several recent studies have demonstrated the advantages of the use of SMRT sequencing technology [44–46]. The methylated motifs detected were all shown to represent adenine-specific methylation. Genome-wide motif analysis resulted in the identification of several MTase recognition motifs with the same motifs detected on two separate sequencing runs (where phages had been propagated on different strains). For both the Phi93 and Phi145 genomes, four distinctly different methylation motifs, 5′-CCC^6m^A-3′, 5′-GT^6m^AG-3′, 5′-CY^6m^AG-3′ and 5′-GGW^6m^AG-3′ (W = A or T, Y = C or T, R = A or G), were identified in the SMRT sequencing data. In addition, the methylation motif 5′-G^6m^ATC-3′was identified for the Phi93 genome, consistent with the presence of *mtPhi93-dam*, which specifies a predicted DAM MTase (predicted to methylate the adenine base in the sequence GATC). This methylation motif was, as expected, not identified on the genome of either Phi15 or Phi145, which do not harbour a predicted DAM-specific (orphan) MTase. Finally, a single methylation motif identified on the Phi15 genome, 5′-CC^6m^AG-3′, was identified on both SMRT sequencing runs. As a result, it is tempting to assign this motif to the presumed methylation activity of the gene product of *mtPhi15-1*, which is located within the replication region of Phi15. A very similar motif was identified for Phi93 and Phi145, 5′-CY^6m^AG-3′ (Y = C or T), which is consistent with the high level of sequence similarity between the gene products of *mtPhi145-2, mtPhi93-2,* and *mtPhi15-1* (Figure 3) and which indicates that the replication module-associated MTases of these three phages are responsible for the 5′-CC^6m^AG-3′ motif methylation.

### MTases protect phage genomes from endonuclease activity

**Figure 4:**
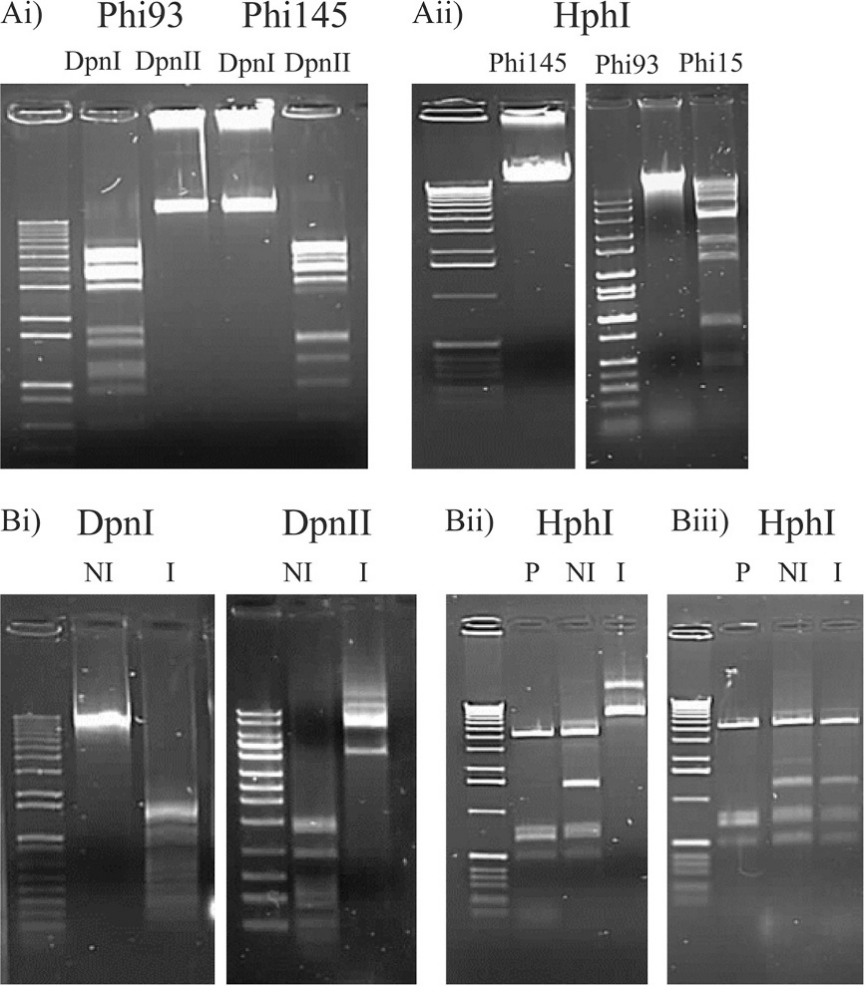
**DNA restriction analyses. A) i)** Phi93 and Phi145 (DAM negative) cut with Dpn1 (cuts methylated GATC) and DpnII (cuts unmethylated GATC). **ii)** Phi145, Phi93 (MTase positive) and Phi15 (MTase negative) with HphI. **B) i)** Plasmid pPiM.93DAM cut with Dpn1 (cuts methylated GATC) and DpnII (cuts unmethylated GATC) under induced (I) and un-induced (NI) conditions. **ii)** Plasmid pPTPi (P), pPiM.145I cut with HphI under induced (I) and un-induced (NI) conditions. **iii)** Plasmid pPTPi (P), pPiM.145II cut with HphI under induced (I) and un-induced (NI) conditions.

To determine whether the putative MTases encoded by the phage genomes provide a protective effect, restriction endonucleases were used to determine if their activity was blocked by active methylation of phage DNA. The 5′-GATC-3′ specific enzymes DpnII (cuts unmethylated DNA) and DpnI (only cuts methylated DNA) were used to determine if the product of *mtPhi93-DAM* is indeed capable of protection of Phi93 genomic DNA against restriction that targets a GATC recognition sequence. As expected, Phi93 genome DNA was protected from restriction by DpnII, while no such protection was observed against DpnI (Figure 4 Ai). In contrast, genomic DNA of Phi145, which is not DAM methylated (Table 3), exhibited the opposite endonuclease-mediated pattern whereby the DNA was restricted by DpnII and not by DpnI (Figure 4 Ai).

The methylation-sensitive, 5′-GGTGA-3′-recognizing restriction enzyme HphI, which exhibits an overlapping target recognition sequence with the methylation motif 5′-GGWG^6m^A-3′ (where W represents either an A or a T; and found in the genomes of Phi93 and Phi145), was utilised to demonstrate that the protein products of *mtPhi145-1* and *mtPhi93-1* protected genomic DNA of phages Phi145 and Phi93 against HphI-mediated restriction. As expected, Phi145 and Phi93 phage DNA was not digested by HphI, while DNA Phi15 was clearly digested by this enzyme (Figure 4 Aii).

To unambiguously link predicted MTase-encoding genes to a specific methylation motif found on the investigated phage genomes, *mtPhi93-DAM*, *mtPhi145-1* and *mtPhi145-2* were individually cloned into the low copy plasmid pPTPi and heterologous expression studies were performed using the nisin-inducible system (*L. lactis* NZ9000 background) to determine if their encoded products had the ability to methylate plasmid DNA and protect against restriction. The genomes of Phi93 and Phi145 contain identical genes encoding putative (orphan) MTases (i.e. the gene products of *mtPhi145-1* and *mtPhi145-2,* are 99% and 100% identical to those of *mtPhi93-1* and *mtPhi93-2*, respectively), therefore the Phi145-associated genes and their encoded products, M.Phi145I and M.Phi145II, were used as representatives for these phage-associated MTases.

pPTPi derivatives were constructed to generate pPiM.93DAM (harbouring gene *mtPhi93-DAM*), pPiM145.1 (harbouring gene *mtPhi145-1*) and pPiM145.1 (harbouring gene *mtPhi145-2*) under the control of a nisin inducible promoter. Following the growth of NZ9000 harbouring pPiM.93DAM with and without nisin, plasmid DNA was restricted with both DpnI and DpnII. pPiM.93DAM DNA isolated from NZ9000 following nisin induction was protected from digestion by DpnII, but restricted by DpnI. The opposite effect was observed under conditions without nisin induction where plasmid DNA was shown to be digested by DpnII and not by DpnI. This shows that the plasmid-located GATC sites were methylated by the expressed gene product of *mtPhi93-DAM* and thus protected against digestion by DpnII (Figure 4 Bi).

Using a similar approach, it was hypothesised that if either of the MTases encoded by the Phi145 genome is associated with the methylation motifs mentioned above, it would protect this phage from HphI digestion. Along with plasmid DNA from the empty vector, pPTPi, plasmid DNA isolated from *L. lactis* NZ9000 strains, harbouring either pPiM145.1 or pPiM145.2, and grown in the presence or absence of nisin was restricted with HphI. Restriction endonuclease digestions showed that DNA of plasmid pPiM145.1 isolated from NZ9000 grown in the presence of nisin was protected from cleavage by HphI, whereas such DNA was not protected when isolated from the same strain grown in the absence of nisin (Figure 4 Bii). The empty vector was digested, as expected, by HphI. Since plasmid DNA of pPiM145.2 isolated from NZ9000 following growth with and without nisin was not resistant to HphI cleavage, (Figure 4 Biii), it is tempting to ascribe the non-palindromic methylome motif, ’-GGWG^6m^A-3′ to the activity of the gene product of *mtPhi145-1* and by default, to that of *mtPhi93-1*.

## Conclusions

To our knowledge, this is the first reported use of SMRT sequencing technology to identify MTases encoded by phage genomes and the first identification of functional MTases associated with the lactococcal 936-type phages (summarized in Table 3). The protective effects provided by these proteins indicate that these particular isolates have aquired these MTase-encoding genes as an enhanced fitness mechanism. The phages were isolated from an undefined mixed starter culture environment (containing 40+ bacterial strains), which may harbour an extensive array of R-M systems. Due to the selection pressure being imposed on infecting phages by such systems, phages may have aquired these methyltransferases to defend themselves from host-encoded R-Ms, a trait not previously observed in 936-type dairy phages. Developments in the SMRT sequencing platform and analysis tools has permitted a novel approach to defining methylation sites within phage and bacterial genomes and in this study has complemented traditional approaches to defining methylation activity. The acquisition of such genetic elements highlights the ever-changing nature and plasticity of these phage genomes and warrants continued genome sequence analysis of phages as novel genetic elements continue to emerge and enhance our understanding of phage evolutionary processes.

## Methods

### Bacterial strains, plasmids and phages

Bacterial cultures, plasmids, phages and primers used in this study are listed in Table 1. Phages were propagated on their respective hosts as described previously [23] and resulting phage lysates were maintained (10 mL) at a titre of approximately 10^8–10^ PFU (*p*laque-*f*orming *u*nits) mL^−1^ at 4°C. *L. lactis* cultures were routinely grown in M17 broth (Oxoid, Hampshire, United Kingdom) supplemented with 0.5% w/v lactose (LM17) or glucose (GM17) at 30°C. *E. coli* strains were routinely grown in Luria Bertani (LB) broth. Growth medium (LM17, GM17 or LB) for strains harbouring plasmid pPTPi or its derivatives were supplemented with 10 μg mL^−1^ tetracycline (Sigma, Co. Wicklow, Ireland) for plasmid maintenance.

### Whole genome sequencing

An equal volume of RNase and DNase-treated, CsCl-purified phage preparation was added to an equal volume of disruption buffer (prepared by the addition of 7.2 μL 2-mercaptoethanol to 1 mL of GTC stock solution [22.5 mL 6 M guanidium thiocyanate solution (Sigma), 6.8 mL H_2_O, 1.76 mL sodium citrate (0.75 M), pH 7 and 2.64 mL 10% sarkosyl]). Following a 30 min incubation at room temperature, an equal volume of phenol:chloroform:isoamyl alcohol (25:24:1) (Sigma) was added, mixed and subjected to centrifugation at 12,300 x g for 5 min. This extraction was repeated, after which DNA present in the aqueous phase was precipitated by the addition of 2.5 volumes 96% ice-cold ethanol and 0.1 volume sodium acetate (pH 4.8) and collected by centrifugation at 12,300 × g for 15 min. The obtained pellet was gently washed in 70% ethanol, allowed to air-dry and finally resuspended in 50–65 μL of TE buffer [23]. Whole genome sequencing was conducted by Macrogen Inc (Korea) using a GS-FLX Titanium sequencer. An average 233-fold sequencing coverage was obtained using pyrosequencing technology on a 454 FLX instrument. The files generated by the 454 FLX instrument were assembled *de novo* with GSassembler (454 Lifesciences, Branford, CT). To ensure correct assembly and to resolve any remaining base conflicts, selected regions of the phage genomes were amplified by PCR and subjected to Sanger sequencing (performed by MWG, Ebersberg, Germany).

### Phage genome annotations

Open reading frames (ORFs) were automatically predicted using the Heuristic Approach for Gene Prediction [47]. Protein-encoding regions with a minimum size of thirty amino acids were selected and the annotation manually curated by predicting the ribosomal binding site and the start and stop codons using the visualization software, Artemis (v15.0.0.1) [48]. BlastP was employed to provide preliminary functional annotation data. Putative (orphan) MTases were identified using REBASE [49] and HHpred [50] and comparative analysis was performed using the MegAlign program of the DNASTAR software package (DNASTAR, Madison, WI, USA).

### SMRT DNA sequencing for methylome analysis

To investigate the methylation sites of phage-encoded (orphan) MTases, 10 mg mL^−1^ of phage DNA (a total of 10 mg) was prepared as above; phage Phi93 was propagated on *L. lactis* strain SM M. To take host-specified methylation activities into account, DNA was isolated from Phi145, which had been propagated on strain SM M or strain SM E (strains were previously shown to have different plasmid profiles and phage infection profiles [23]), while Phi15 was propagated on strain SM 11 or strain SM 13. Whole genome sequencing was performed on a Pacific Biosciences RS2 machine with C2/P4 chemistry at the Functional Genomics Centre, Zurich. The results were analysed with SMRTanalysis 2.0 software (http://pacbiodevnet.com/) using protocol “RS_Motification_and_Motif_Analysis.1” with default settings. For the detection of some methylation patterns, the Minimum Modification QV set to the more stringent setting of 60. The library was prepared according to the manufacturer’s instructions (PacBio, Germany).

### MTase cloning

For the construction of pPTPi derivative plasmids, pPiM.93DAM, pPiM.145I and pPiM.145II, DNA fragments encompassing the coding sequences of *mtPhi93-dam* (corresponding to locus tag Phi93_04), *mtPhi145-1* and *mtPhi145-2* (corresponding to locus tags Phi145_02 and Phi145_37, respectively) were generated by PCR amplification using the primers listed in Table 1 and employing KOD high fidelity polymerase (Millipore, Cork, Ireland). Each of these amplicons was cloned into pPTPi, using *E. coli* as a cloning host (*DAM*^*−*^/*DCM*^*−*^ K12 for *mtPhi93-dam*, and EC101 for *mt1451* and *mt1452*) and selected on LB agar plates supplemented with 10 μg mL^−1^ tetracycline at 37°C. Sanger sequencing was employed to verify the integrity of each of the generated constructs (MWG Eurofins, Germany) using relevant plasmid-associated primers (Table 1) [51].

### MTases protein expression, plasmid isolation and DNA restriction

Plasmid constructs were isolated from *E. coli* and transformed into *L. lactis* NZ9000 for protein expression using the Nisin-Inducible Expression System (NICE) [52]. *L. lactis* NZ9000 harboring pPiM.93DAM, pPiM.145I and pPiM.145II were grown overnight at 30°C in GM17 broth supplemented with tetracycline at 10 μg mL^−1^. A 2% inoculum of *L. lactis* NZ9000 harboring pPiM.93DAM, pPiM.145I and pPiM.145II overnight bacterial cultures was transferred into fresh 10 mL GM17 containing 5 μg mL^−1^ tetracycline and incubated at 30°C. When the optical density at 600 nm had reached 0.2, protein expression was induced by the addition of Nisaplin™ at a concentration of 100 ng mL^−1^. Un-induced controls were incubated as above without the addition of Nisaplin™. Following a 3 h incubation at 30°C, the induced cells harboring pPiM.93DAM, pPiM.145I and pPiM.145II were harvested by centrifugation (5, 580 × g, 10 min) and subsequently incubated in protoplast buffer (20 mM Tris–HCl, pH 7.5, 5 mM EDTA, 0.75 M sucrose, 10 mg mL^−1^ lysozyme and 50 units mL^−1^ mutanolysin; Sigma) at 37°C for 30 min. Each sample was centrifuged at 1,700 × g for 5 min and plasmid preparations were performed using the GeneJet plasmid miniprep kit as described by the manufacturer (Thermo Scientific, Dublin, Ireland). Restriction endonuclease digests were performed on phage DNA, plasmid DNA and bacterial genomic DNA using DpnI and DpnII (Roche, United States), or HphI (NEB, United States), all according to the manufacturer’s instructions.

### Nucleotide sequence accession numbers

All the sequences generated have been submitted to GenBank database with the following accession numbers: Phi15 [GenBank: KM091442], Phi93 [GenBank: KM091443] and Phi145 [GenBank: KM091444].

## Electronic supplementary material

Additional file 1: Table S1: Putative predicted ORFs of the 936-type phage. A table containing a list of the ORFs, corresponding genomic coordinates and predicted function of Phi93, Phi15 and Phi145. (DOCX 27 KB)

## References

[1] Stern A, Sorek R (2011). The phage-host arms race: shaping the evolution of microbes. Bioessays.

[2] Gomez P, Buckling A (2011). Bacteria-phage antagonistic coevolution in soil. Science.

[3] Weitz JS, Hartman H, Levin SA (2005). Coevolutionary arms races between bacteria and bacteriophage. Proc Natl Acad Sci U S A.

[4] Wei Y, Ocampo P, Levin BR (2010). An experimental study of the population and evolutionary dynamics of *Vibrio cholerae* O1 and the bacteriophage JSF4. Proc Biol Sci.

[5] Bohannan BJM, Lenski RE (2000). Linking genetic change to community evolution: insights from studies of bacteria and bacteriophage. Ecol Lett.

[6] Chatterjee S, Rothenberg E (2012). Interaction of bacteriophage l with its *E. coli* receptor, LamB. Viruses.

[7] Meyer JR, Dobias DT, Weitz JS, Barrick JE, Quick RT, Lenski RE (2012). Repeatability and contingency in the evolution of a key innovation in phage lambda. Science.

[8] Labrie SJ, Samson JE, Moineau S (2010). Bacteriophage resistance mechanisms. Nat Rev Microbiol.

[9] Szczepankowska A (2012). Role of CRISPR/cas system in the development of bacteriophage resistance. Adv Virus Res.

[10] Deveau H, Barrangou R, Garneau JE, Labonte J, Fremaux C, Boyaval P, Romero DA, Horvath P, Moineau S (2008). Phage response to CRISPR-encoded resistance in *Streptococcus thermophilus*. J Bacteriol.

[11] Murphy J, Mahony J, Ainsworth S, Nauta A, van Sinderen D (2013). Bacteriophage orphan DNA methyltransferases: insights from their bacterial origin, function, and occurrence. Appl Environ Microbiol.

[12] Kossykh VG, Schlagman SL, Hattman S (1995). Phage T4 DNA [N6-adenine]methyltransferase. Overexpression, purification, and characterization. J Biol Chem.

[13] Hill C, Miller LA, Klaenhammer TR (1991). In vivo genetic exchange of a functional domain from a type II A methylase between lactococcal plasmid pTR2030 and a virulent bacteriophage. J Bacteriol.

[14] Günthert U, Lauster R, Reiners L (1986). Multispecific DNA methyltransferases from *Bacillus subtilis* phages. E J Biochem.

[15] Dempsey RM, Carroll D, Kong H, Higgins L, Keane CT, Coleman DC (2005). Sau42I, a BcgI-like restriction-modification system encoded by the *Staphylococcus aureus* quadruple-converting phage π42. Microbiology.

[16] Coakley M, Fitzgerald G, Ros RP (1997). Application and evaluation of the phage resistance- and bacteriocin-encoding plasmid pMRC01 for the improvement of dairy starter cultures. Appl Environ Microbiol.

[17] Murphy J, Mahony J, van Sinderen D (2014). Impact of thermal and biocidal treatments on lactococcal 936-type phages. Int Dairy J.

[18] Campagna C, Villion M, Labrie SJ, Duchaine C, Moineau S (2014). Inactivation of dairy bacteriophages by commercial sanitizers and disinfectants. Int J Food Microbiol.

[19] Bouchard JD, Moineau S (2004). Lactococcal phage genes involved in sensitivity to AbiK and their relation to single-strand annealing proteins. J Bacteriol.

[20] Boucher I, Emond E, Dion E, Montpetit D, Moineau S (2000). Microbiological and molecular impacts of AbiK on the lytic cycle of *Lactococcus lactis* phages of the 936 and P335 species. Microbiology.

[21] Haaber J, Rousseau GM, Hammer K, Moineau S (2009). Identification and characterization of the phage gene sav, involved in sensitivity to the lactococcal abortive infection mechanism AbiV. Appl Environ Microbiol.

[22] Ainsworth S, Mahony J, van Sinderen D (2014). The plasmid complement of *Lactococcus lactis* UC509. 9 encodes multiple bacteriophage resistance systems. Appl Environ Microbiol.

[23] Murphy J, Royer B, Mahony J, Hoyles L, Heller K, Neve H, Bonestroo M, Nauta A, van Sinderen D (2013). Biodiversity of lactococcal bacteriophages isolated from 3 Gouda-type cheese-producing plants. J Dairy Sci.

[24] Josephsen J, Petersen A, Neve H, Nielsen EW (1999). Development of lytic *Lactococcus lactis* bacteriophages in a Cheddar cheese plant. Int J Food Microbiol.

[25] Rousseau GM, Moineau S (2009). Evolution of *Lactococcus lactis* phages within a cheese factory. Appl Environ Microbiol.

[26] Mahony J, Deveau H, Mc Grath S, Ventura M, Canchaya C, Moineau S, Fitzgerald GF, van Sinderen D (2006). Sequence and comparative genomic analysis of lactococcal bacteriophages jj50, 712 and P008: evolutionary insights into the 936 phage species. FEMS Microbiol Lett.

[27] Klumpp J, Fouts DE, Sozhamannan S (2012). Next generation sequencing technologies and the changing landscape of phage genomics. Bacteriophage.

[28] Castro-Nallar E, Chen H, Gladman S, Moore SC, Seemann T, Powell IB, Hillier A, Crandall KA, Chandry PS (2012). Population genomics and phylogeography of an Australian dairy factory derived lytic bacteriophage. Genome Biol Evol.

[29] Roberts RJ, Belfort M, Bestor T, Bhagwat AS, Bickle TA, Bitinaite J, Blumenthal RM, Degtyarev SK, Dryden DT, Dybvig K (2003). A nomenclature for restriction enzymes, DNA methyltransferases, homing endonucleases and their genes. Nucleic Acids Res.

[30] Malone T, Blumenthal RM, Cheng X (1995). Structure-guided analysis reveals nine sequence motifs conserved among DNA amino-methyl-transferases, and suggests a catalytic mechanism for these enzymes. J Mol Biol.

[31] Bheemanaik S, Bujnicki JM, Nagaraja V, Rao DN (2006). Functional analysis of amino acid residues at the dimerisation interface of KpnI DNA methyltransferase. Biol Chem.

[32] Murphy J, Mahony J, van Sinderen D (2013). Complete genome sequence of the 936-type lactococcal bacteriophage *Caseus*JM1. Genome Announc.

[33] Yang Z, Horton JR, Zhou L, Zhang XJ, Dong A, Zhang X, Schlagman SL, Kossykh V, Hattman S, Cheng X (2003). Structure of the bacteriophage T4 DNA adenine methyltransferase. Nat Struct Biol.

[34] Coren JS, Pierce JC, Sternberg N (1995). Headful packaging revisited: the packaging of more than one DNA molecule into a bacteriophage P1 head. J Mol Biol.

[35] Canchaya C, Fournous G, Chibani-Chennoufi S, Dillmann M-L, Brüssow H (2003). Phage as agents of lateral gene transfer. Curr Opin Microbiol.

[36] Quiles-Puchalt N, Carpena N, Alonso JC, Novick RP, Marina A, Penadés JR (2014). Staphylococcal pathogenicity island DNA packaging system involving cos-site packaging and phage-encoded HNH endonucleases. Proc Natl Acad Sci U S A.

[37] Nyengaard N, Vogensen FK, Josephsen J (1995). Restriction-modification systems in *lactococcus lactis*. Gene.

[38] Moineau S, Walker SA, Vedamuthu ER, Vandenbergh PA (1995). Cloning and sequencing of LlaDCHI [corrected] restriction/modification genes from *Lactococcus lactis* and relatedness of this system to the *Streptococcus pneumoniae* DpnII system. Appl Environ Microbiol.

[39] Tran-Betcke A, Behrens B, Noyer-Weidner M, Trautner T (1986). DNA methyltransferase genes of *Bacillus subtilis* phages: comparison of their nucleotide sequences. Gene.

[40] Sternberg N, Coulby J (1990). Cleavage of the bacteriophage P1 packaging site (pac) is regulated by adenine methylation. Proc Natl Acad Sci U S A.

[41] Eid J, Fehr A, Gray J, Luong K, Lyle J, Otto G, Peluso P, Rank D, Baybayan P, Bettman B (2009). Real-time DNA sequencing from single polymerase molecules. Science.

[42] Flusberg BA, Webster DR, Lee JH, Travers KJ, Olivares EC, Clark TA, Korlach J, Turner SW (2010). Direct detection of DNA methylation during single-molecule, real-time sequencing. Nat Methods.

[43] Dahl C, Guldberg P (2003). DNA methylation analysis techniques. Biogerontology.

[44] Fang G, Munera D, Friedman DI, Mandlik A, Chao MC, Banerjee O, Feng Z, Losic B, Mahajan MC, Jabado OJ (2012). Genome-wide mapping of methylated adenine residues in pathogenic *Escherichia coli* using single-molecule real-time sequencing. Nat Biotechnol.

[45] Bendall ML, Luong K, Wetmore KM, Blow M, Korlach J, Deutschbauer A, Malmstrom RR (2013). Exploring the roles of DNA methylation in the metal-reducing bacterium *Shewanella oneidensis* MR-1. J Bacteriol.

[46] Krebes J, Morgan RD, Bunk B, Spröer C, Luong K, Parusel R, Anton BP, König C, Josenhans C, Overmann J (2013). The complex methylome of the human gastric pathogen *Helicobacter pylori*. Nucleic acids Res.

[47] Besemer J, Borodovsky M (1999). Heuristic approach to deriving models for gene finding. Nucleic Acids Res.

[48] Rutherford K, Parkhill J, Crook J, Horsnell T, Rice P, Rajandream M-A, Barrell B (2000). Artemis: sequence visualization and annotation. Bioinformatics.

[49] Roberts RJ, Vincze T, Posfai J, Macelis D (2010). REBASE—a database for DNA restriction and modification: enzymes, genes and genomes. Nucleic Acids Res.

[50] Söding J, Biegert A, Lupas AN (2005). The HHpred interactive server for protein homology detection and structure prediction. Nucleic Acids Res.

[51] Collins B, Bebeacua C, Mahony J, Blangy S, Douillard FP, Veesler D, Cambillau C, van Sinderen D (2013). Structure and functional analysis of the host recognition device of lactococcal phage Tuc 2009. J Virol.

[52] Douillard FP, Mahony J, Campanacci V, Cambillau C, van Sinderen D (2011). Construction of two *Lactococcus lactis* expression vectors combining the Gateway and the Nisin Controlled Expression systems. Plasmid.

[53] Linares DM, Kok J, Poolman B (2010). Genome sequences of *Lactococcus lactis* MG1363 (revised) and NZ9000 and comparative physiological studies. J Bacteriol.

[54] Law J, Buist G, Haandrikman A, Kok J, Venema G, Leenhouts K (1995). A system to generate chromosomal mutations in *Lactococcus lactis* which allows fast analysis of targeted genes. J Bacteriol.

