# Supplementary material for: Methyltransferases acquired by lactococcal 936-type phage provide protection against restriction endonuclease activity
Source: BMC Genomics. 2014 Oct 1;15(1):831. doi: 10.1186/1471-2164-15-831 (PMC4190342; doi:10.1186/1471-2164-15-831)
Supplement: Supplementary file 1 — Additional file 1: Table S1: Putative predicted ORFs of the 936-type phage. A table containing a list of the ORFs, corresponding genomic coordinates and predicted function of Phi93, Phi15 and Phi145. (DOCX 27 KB) [file 12864_2014_6505_MOESM1_ESM.docx]

**Table S1 Putative predicted ORFs of the 936-type phages.**

| **Phi93** | | | **Phi15** | | | **Phi145** | | |
| --- | --- | --- | --- | --- | --- | --- | --- | --- |
| ***orf*** | **Coordinates** | **Protein** | ***orf*** | **Coordinates** | **Protein** | ***orf*** | **Coordinates** | **Protein** |
| 1 | 260-784 | small terminase | 1 | 232-756 | Small terminase | 1 | 263-787 | Small terminase |
| 2 | 781-1629 | DNA MTase | 2 | 753-1214 | HNHE | 2 | 784-1641 | DNA MTase |
| 3 | 1684-2247 | HP^a^ | 3 | 1225-2838 | Large terminase | 3 | 1653-3275 | Large terminase |
| 4 | 2368-2790 | DNA MTase | 4 | 2828-3112 | HNHE | 4 | 3265-3549 | HNHE |
| 5 | 2820-4424 | Large terminase | 5 | 3125-4261 | Portal Protein | 5 | 3580-4698 | Portal Protein |
| 6 | 4414-4698 | HNHE^b^ | 6 | 4242-4778 | Protease | 6 | 4679-5215 | Protease |
| 7 | 4711-5847 | Portal Protein | 7 | 4771-5952 | Minor Structural | 7 | 5208-6389 | Minor structural |
| 8 | 5828-6364 | Protease | 8 | 5973-6236 | HSP1 | 8 | 6410-6673 | HSP1 |
| 9 | 6357-7538 | Minor Structural | 9 | 6237-6550 | HSP2 | 9 | 6673-6987 | HSP2 |
| 10 | 7559-7822 | HSP ^c^ 1 | 10 | 6547-6888 | HSP3 | 10 | 6984-7481 | HNHE |
| 11 | 7822-8136 | HSP2 | 11 | 6879-7244 | HSP4 | 11 | 7471-7809 | HSP3 |
| 12 | 8133-8630 | HNHE | 12 | 7302-9890 | Neck passage structure | 12 | 7800-8165 | HSP4 |
| 13 | 8620-8958 | HSP3 | 13 | 10062-10967 | Major Capsid | 13 | 8178-9083 | Major Capsid |
| 14 | 8949-9314 | HSP4 | 14 | 11006-11281 | HSP5 | 14 | 9169-9708 | Accessory fiber |
| 15 | 9327-10232 | Major Capsid | 15 | 11301-11813 | HSP6 | 15 | 9728-10003 | HSP5 |
| 16 | 10318-10857 | Accessory fiber | 16 | 11813-14323 | Tail tape measure | 16 | 10023-10535 | HSP6 |
| 17 | 10877-11152 | HSP5 | 17 | 14323-15219 | HSP7 | 17 | 10535-13534 | Tail tape measure |
| 18 | 11172-11684 | HSP6 | 18 | 15219-16346 | HSP8 | 18 | 13534-15009 | HSP7 |
| 19 | 11684-14683 | Tail tape measure | 19 | 16336-16629 | HSP9 | 19 | 15009-16136 | HSP8 |
| 20 | 14683-16158 | HSP7 | 20 | 16619-17380 | Receptor binding | 20 | 16126-16419 | HSP9 |
| 21 | 16158-17285 | HSP8 | 21 | 17402-17755 | Holin | 21 | 16409-17215 | Receptor binding |
| 22 | 17275-17568 | HSP9 | 22 | 17752-18582 | Lysin | 22 | 17229-17582 | Holin |
| 23 | 17558-18364 | Receptor binding | **23 ^d^** | 18710-19075 | HNHE | 23 | 17579-18295 | Lysin |
| **Phi93** | | | **Phi15** | | | **Phi145** | | |
| ***orf*** | **Coordinates** | **Protein** | ***orf*** | **Coordinates** | **Protein** | ***orf*** | **Coordinates** | **Protein** |
| 24 | 18378-18731 | Holin | **24** | 19104-19631 | HP | **24 ^d^** | 18341-18505 | HP |
| 25 | 18728-19444 | Lysin | **25** | 19741-20010 | HP | **25** | 18547-18804 | HP |
| **26^d^** | 19490-19654 | HP | **26** | 20007-2018 | HP | **26** | 18833-19081 | HP |
| **27** | 19696-19953 | HP | **27** | 20183-20530 | HP | **27** | 19102-19368 | HP |
| **28** | 19985-20233 | HP | **28** | 20527-20697 | HP | **28** | 19369-19518 | HP |
| **29** | 20254-20520 | HP | **29** | 20697-20873 | HP | **29** | 19517-19617 | HP |
| **30** | 20521-20670 | HP | **30** | 20866-21102 | HP | **30** | 19692-20039 | HP |
| **31** | 20671-20769 | HP | **31** | 21165-21410 | HP | **31** | 20039-20272 | HP |
| **32** | 20844-21191 | HP | **32** | 21407-21673 | HP | **32** | 20384-20575 | HP |
| **33** | 21191-21424 | HP | **33** | 21753-22061 | HP | **33** | 20617-20874 | HP |
| **34** | 21536-21727 | HP | **34** | 22061-22180 | HP | **34** | 21050-21511 | HP |
| **35** | 21770-22027 | HP | **35** | 22186-22848 | HP | **35** | 21483-21788 | HP |
| **36** | 22203-22664 | HP | **36** | 22904-23806 | DNA MTase | **36** | 21789-21908 | HP |
| **37** | 22636-22941 | HP | **37** | 23917-24339 | HNHE | **37** | 21966-22979 | DNA MTase |
| **38** | 22942-23061 | HP | **38** | 24340-24450 | HP | **38** | 22972-23076 | HP |
| **39** | 23119-24132 | DNA MTase | **39** | 24447-24620 | HP | **39** | 23299-23808 | HP |
| **40** | 24125-24373 | HP | **40** | 24703-25212 | HP | **40** | 23805-24020 | HP |
| **41** | 24452-24961 | HP | **41** | 25209-25424 | HP | **41** | 24033-24392 | SSB |
| **42** | 24958-25173 | HP | **42** | 25437-25703 | SSB | **42** | 24396-24950 | SAK Kinase |
| **43** | 25186-25545 | SSB | **43** | 25802-26128 | SAK Kinase | **43** | 24947-25057 | HNHE |
| **44** | 25549-26103 | SAK Kinase | **44** | 26364-26864 | HNHE | **44** | 25044-25415 | HP |
| **45** | 26100-26210 | HNH Endonuclease | **45** | 26851-26976 | HP | **45** | 25551-25940 | HP |
| **46** | 26197-26610 | HP | **46** | 26963-27349 | HP | **46** | 25997-26254 | HP |
| **47** | 26704-27093 | HP | **47** | 27473-27859 | HP | **47** | 26247-27197 | DNA Polymerase |
| **48** | 27150-27407 | DNA Polymerase | **48** | 27910-28173 | HP | **48** | 27291-27518 | HP |
|  |  |  |  |  |  |  |  |  |
| **Phi93** | | | **Phi15** | | | **Phi145** | | |
| ***orf*** | **Coordinates** | **Protein** | ***orf*** | **Coordinates** | **Protein** | ***orf*** | **Coordinates** | **Protein** |
| **49** | 27400-28419 | HP | **49** | 28166-29116 | DNA Polymerase | **49** | 27560-28240 | HP |
| **50** | 28444-28671 | HP | **50** | 29843-29971 | HP | **50** | 28676-28865 | HP |
| **51** | 29166-29285 | HP | **51** | 30341-30529 | HP | 51 | 28995-29123 | M1 |
| **52** | 29640-29828 | M1 | 52 | 30660-30788 | M1 | 52 | 29128-29259 | M2 |
| 53 | 29958-30086 | M2 | 53 | 30793-30924 | M2 | 53 | 29256-29738 | Holiday junction endonuclease |
| 54 | 30091-30222 | Holiday junction endonuclease | 54 | 30921-31400 | Holiday junction endonuclease | 54 | 29735-29878 | M4 |
| 55 | 30219-30701 | M4 | 55 | 31401-31562 | M4 | 55 | 29896-30063 | M5 |

^a^ HP=hypothetical

^b^ HNHE= HNH endonuclease

^c^ Hypothetical structural protein

^d^ Bold numbers indicate ORFs on the reverse strand
